# Ocular Factors Affecting AI Diagnosis in Diabetic Retinopathy Screening for Resource-Limited Regions: Cross-Sectional Study

**DOI:** 10.2196/85181

**Published:** 2026-09-30

**Authors:** Ying Xue, Xin Hu, Shuaijie Yuan, Junfang Zhang, Hongxia Hu, Shangbo Yang, Rongrong Zhu

**Affiliations:** 1 Department of Ophthalmology, Affiliated Hospital of Nantong University, 20 Xisi Road, Chongchuan District, Nantong City, Jiangsu Province, China, Nantong, 226001, China, 86 85052222, 86 85052222; 2 Department of Ophthalmology, Funing Shizhuang Eye Hospital, Yancheng, Yanchen, China; 3 Funing County Center for Disease Prevention and Control, Yanchen, China

**Keywords:** diabetic retinopathy, artificial intelligence, AI, telemedicine, screening, diagnostic performance

## Abstract

**Background:**

Diabetic retinopathy (DR) is the leading cause of vision loss among working-age adults worldwide. AI-assisted automated image reading has effectively alleviated the human resource challenges in large-scale remote screenings, yet there is limited analysis on the impact of complex ocular factors on the diagnostic efficacy of AI.

**Objective:**

This study aimed to systematically analyze the impact of multiple ocular factors on the diagnostic efficacy of the EVisionAI system in detecting DR and vision-threatening diabetic retinopathy (VTDR) during large-scale screening of residents with diabetes in resource-limited regions.

**Methods:**

This cross-sectional study used a multistage stratified random sampling method to screen residents with type 2 diabetes at primary health centers in resource-limited regions. Data collection involved structured questionnaires (for basic and disease information), hemoglobin A_1c_ testing, and comprehensive ophthalmic examinations (visual acuity, intraocular pressure, slit-lamp examination, axial length measurement, and fundus photography). Following data collection, 2 ophthalmologists independently graded fundus photographs according to the American Academy of Ophthalmology standards. The influence of various ocular factors on the diagnostic performance of EVisionAI was subsequently evaluated.

**Results:**

Between October 21 and November 12, 2024, 1847 participants with type 2 diabetes (aged 32‐91 years) were enrolled, of whom 1748 (94.6%) completed the screening process and 3392 eyes were eligible for DR analysis. Participants had a mean age of 67.17 (SD 8.52) years and mean diabetes duration of 9.13 (SD 6.97) years, with 19.2% (335/1748) having DR and 7.4% (129/1748) having VTDR. Although EVisionAI’s diagnostic efficacy was comparable to that of ophthalmologists (sensitivity: 90.61%, 95% CI 87.89%-92.79%; specificity: 98.99%, 95% CI 98.52%-99.31%), some ocular factors—including pupil size, refractive media opacity, and tessellated fundus (TF)—significantly impaired its efficiency. Severe refractive media opacity and TF reduced its sensitivity to 80.95% and 82.86%, respectively, and these factors interfered more with early-stage DR detection than VTDR (97.45% detected), particularly in eyes with severe TF changes (sensitivity decreased to 60.71%). Most notably, severe vitreous degeneration-induced opacity almost invariably led to VTDR misdiagnosis. Additionally, pupil dilation improved the sensitivity of EVisionAI for diagnosing DR (excluding early-stage DR) but had minimal impact on specificity.

**Conclusions:**

EVisionAI achieved high diagnostic accuracy for large-scale DR screening in resource-limited regions, yet its performance for early-stage disease was diminished by severe ocular factors. Optimizing for these factors is therefore essential to maximize its clinical utility in primary care settings with limited specialist access.

## Introduction

Diabetes is one of the biggest health care challenges in the world [1]. The prevalence of diabetes in adults aged 20 to 79 years is expected to increase from approximately 537 million in 2021 to 783 million by 2045 [2]. Diabetic retinopathy (DR) is the leading cause of vision loss in working-age adults worldwide [3-5]. About 35% of patients with diabetes are at risk of developing DR, with more than 10% at risk of more severe vision-threatening DR (VTDR, including severe nonproliferative diabetic retinopathy [NPDR] and proliferative diabetic retinopathy) [5,6]. Vision loss from DR may occur asymptomatically in the early stage and rapidly develop into VTDR without awareness and intervention, resulting in irreversible visual damage [6]. Nevertheless, blindness caused by DR is still considered “avoidable blindness”; regular surveillance by clinical examination or grading of retinal photographs can help identify vision-threatening retinopathy [5]. Early-stage interventions via glycemia and blood pressure control can slow the progression of DR, and late-stage interventions through photocoagulation or intravitreal injection can reduce vision loss [7]. The optimal time for DR prevention is before vision impairment occurs, but patients rarely seek ophthalmic care proactively at this stage. Indeed, 21% of patients with diabetes worldwide have never undergone DR screening, and in high-income countries only 60% of patients actively receive annual dilated examinations [8,9]. Annual retinal screening is recommended by almost all professional associations; however, due to the shortage of eye care specialists, comprehensive vision screening is rarely completed [4,10,11]. China currently has the largest number of patients with diabetes in the world [12,13], with an approximate ratio of ophthalmologists to patients with diabetes of 1:3000, indicating a significant gap in medical resources [11]. As a cost-effective preventive measure, the state encourages regular DR screening at the community level. However, grassroots health care workers, particularly those serving low-income groups and in resource-limited regions, face the challenges of deficient professional expertise and inadequate training [11]. Therefore, there is an urgent need for diagnostic systems using deep learning algorithms to assist in large-scale DR screening.

The emergence of AI fundus image reading systems has provided technological assurance for large-scale early screening of DR [1,8]. AI-assisted screening systems enable automatic DR detection without requiring professional human graders, offering increased access to eye screening for patients with diabetes—particularly among low-income groups and those in resource-limited regions. This helps address disparities in health care resource allocation and may potentially enhance patients’ compliance with regular ophthalmic examinations [4,14,15]. Early deep learning systems applied to DR screening primarily focused on identifying patients requiring referral (those with moderate NPDR or worse) or patients with VTDR. These systems significantly improved the referral rates for patients with DR and enabled them to receive early diagnosis and treatment services [8,11,16,17]. In recent years, deep learning systems targeting early-stage DR (including mild to moderate NPDR) lesions have also started to emerge. Currently, several AI systems worldwide that assist in fundus disease diagnosis and treatment, such as EyeArt [18] (Eyenuk Inc), IDx-DR [14] (Digital Diagnostics Inc), Retmarker [4] (Retmarker Ltd), DeepDR [11] (Shanghai Diabetes Institute), and Eye Wisdom [19] (Vistel Ltd), all demonstrate high sensitivity and specificity. They have essentially achieved comprehensive, automated diagnosis of DR across the entire disease progression spectrum.

However, although previous studies have shown that AI-assisted DR diagnosis achieves accuracy comparable to that of expert graders, we note significant variability in the sensitivity and specificity of AI diagnosis across different studies and DR populations [1,17]. DR grading itself relies on the overall presence and distribution of DR lesions. However, most AI-assisted DR screening systems perform inference-based assessments using only 1 to 2 field images [20]. In this context, unavoidable ocular interference factors—affecting retinal image quality independent of capture technique or device performance—such as pupil size, refractive media opacity, and TF changes, can significantly impair the AI system’s correct assessment of retinopathy. Our study, based on large-scale AI-assisted DR screening of residents with diabetes in underserved areas, systematically analyzed the impact of multiple ocular factors in the screened population on the efficacy of AI in diagnosing DR and VTDR, providing a foundation for optimizing large-scale automated AI-assisted DR screening.

## Methods

This was a cross-sectional, population-based diagnostic study conducted among rural residents with type 2 diabetes in Funing County, China. A multistage stratified random sampling method was used to recruit patients with type 2 diabetes, and data were collected through structured questionnaires, hemoglobin A_1c_ (HbA_1c_) testing, and comprehensive ophthalmic examinations to evaluate the performance of the EVisionAI automated DR detection system.

### Inclusion and Exclusion Criteria

#### Overview

Funing County has long been ranked in the lower-to-mid economic tier within the province and was therefore selected as the study site. From October 21 to November 12, 2024, eligible participants were selected from the population of patients with type 2 diabetes registered at the Funing County Center for Disease Control and Prevention (CDC).

To initially identify possible participants suitable for recruitment, the Funing County CDC diabetes registry was linked with quarterly population mobility data (routinely updated by the local CDC). Residents meeting the inclusion criteria were retained, while those meeting exclusion criteria were removed. This process generated a sampling frame of potentially eligible participants.

#### Inclusion Criteria

Eligible participants were individuals with diabetes who were registered at the Funing County CDC and were permanent residents of the area or had lived in the area for more than 6 months per year (including migrant populations).

#### Exclusion Criteria

Residents with local household registration who had left the survey area for more than half a year, and residents with severe mental illness who were unable to complete various examinations or provide medical history information were excluded.

### Sampling Method and Sample Size Calculation

This study used a multistage stratified random sampling method. The sample size was calculated using the formula: n=Z^2^ [p (1–p)]/B^2^. At a 95% confidence level, Z=1.96, B=0.15p, and prevalence value (P)=18.2%. The initial sample size was adjusted by a sampling factor of 2.0, resulting in a minimum required survey population of at least 1534 individuals. Accounting for an estimated response rate of 90%, a minimum of 1705 individuals were deemed necessary.

Stratification of townships was performed based on the number of patients with diabetes registered with the Funing County CDC. Townships were stratified using a criterion of 1000 patients with diabetes per stratum. Townships with more than 2000 patients with diabetes were split into 2 or more strata, while those with fewer than 500 patients were combined into 1 stratum. Two townships were then selected using a random number table. Subsequently, villages within these 2 selected townships were further stratified using a criterion of 50 patients with diabetes per stratum. Villages with more than 100 patients with diabetes were counted as 2 or more strata, while those with fewer than 25 patients were combined into 1 stratum. Villages were similarly selected using a random number table.

It should be noted that, when a village was divided into multiple virtual strata based on geographical subunits (eg, streets or residential clusters), these strata were assigned to preserve local spatial information. In the actual sampling for this study, no two strata from the same original village were simultaneously selected, thus avoiding potential within-village clustering in the analysis.

### Research Content

#### Collection of Basic Information

Basic information, including name, gender, age, diabetes history (type, duration, treatment status, and family history), and histories of ocular diseases and ophthalmic surgery, was collected using structured questionnaires administered by trained survey personnel.

#### HbA_1c_ Testing

For HbA_1c_ testing, 2 mL to 3 mL of venous blood was collected into a tube. After labeling with patient identifiers, the sample was analyzed using an automated HbA_1c_ analyzer (TOSOH HLC-723G8).

#### Ophthalmic Examination

Comprehensive ophthalmic examinations included visual acuity assessment using ETDRS charts (including presenting visual acuity [PVA] and best-corrected visual acuity [BCVA] obtained by an optometrist through subjective refraction), intraocular pressure measurement with a TOPCON CT-800 noncontact tonometer (the average of 3 measurements was recorded), slit-lamp examination of the anterior segment (evaluating lens opacity, anterior chamber depth, etc), axial length measurement via a Visionhealthmed Swan 700 biometer prior to pupil dilation, and fundus photography with or without pupil dilation.

### Recruitment Procedure

Participants were notified by telephone to confirm the exact date, time, and screening location (township health centers). Upon arrival, staff at the health centers assisted participants in completing questionnaires covering basic and disease-related information. Nurses then collected blood samples and performed HbA_1c_ testing, with results provided to participants before the conclusion of the screening procedure. Following assessments of PVA and BCVA by optometrists, participants sequentially underwent axial length (AL) and intraocular pressure measurements performed by trained technicians. Subsequently, an ophthalmologist conducted slit-lamp examinations and, when conditions permitted, administered bilateral pupillary dilation. Vitreous degeneration was defined as the presence of vitreous liquefaction, posterior vitreous detachment, or visible vitreous opacities (eg, dust-like, spider web, or diffuse haze) on slit-lamp biomicroscopy. Participants then underwent 2-field digital color fundus photography (those receiving mydriasis waited approximately 20 minutes beforehand). Finally, participants submitted their completed questionnaires, thereby concluding the screening process.

### Fundus Image Acquisition and Grading Standard

#### Overview

Two-field digital color fundus photographs (45° images centered on the optic disc and macula, respectively; RetiCam3100, SYSEYE) were obtained for each eye. In brief, all participants first underwent slit-lamp examination of the anterior segment. Those without contraindications to mydriasis (eg, peripheral anterior chamber depth [ACD] less than 1/2 CT) received compound tropicamide for bilateral mydriasis. Fundus images were then captured after 20 to 30 minutes. These images were subsequently transferred to a cloud-based platform for analysis.

Fundus photograph analysis was performed using the EVisionAI software (Bigvision Medical Technology Co, Ltd) [21,22]. Additional details regarding EVisionAI can be found in Multimedia Appendix 1. This AI system integrates computer vision, deep learning architectures, and advanced image processing technologies, inspired by principles of human visual perception. The software is capable of identifying pathological changes in color fundus photographs associated with myopia, DR, and other ocular conditions.

#### DR Grading

Two-field fundus photographs were diagnosed and graded by 2 independent ophthalmologists according to the 2016 AAO DR grading standards [23]. In cases of disagreement, an internal discussion was conducted to reach a consensus. If consensus could not be achieved, the case was adjudicated by a senior specialist (the corresponding author of this study).

#### Image Clarity Grading

Following the established image quality grading criteria outlined in the literature [16], the acquired 2-field images underwent separate quality assessments for the macular and optic disc regions. The images were graded according to the following 4 levels:

- Grade 0: no problems with any image quality factors; all retinopathy lesions were gradable.
- Grade 1: problem with 1 to 2 image quality factors; all retinopathy lesions were gradable.
- Grade 2: problems with 3 to 4 image quality factors; all retinopathy lesions were gradable.
- Grade 3: one or more retinopathy lesions cannot be graded, but part of the image was gradable.

Images graded as “insufficient for full interpretation” were excluded from further analysis.

#### Fundus Tessellation Grading

In accordance with the protocol established in the literature [24,25], we graded the degree of tessellation in the acquired 2-field fundus images based on the visibility of the choroidal vessels. The images were graded according to the following 4 levels: grade 0 (no tessellation), grade 1 (mild tessellation), grade 2 (moderate tessellation), and grade 3 (marked [severe] tessellation).

Images of inadequate quality for reliable grading were excluded.

### Statistical Analysis

Results for DR and VTDR were analyzed separately in each subgroup. Statistical analyses were performed using SPSS (version 27.0, IBM Corp) and R (version 4.4.2; R Foundation for Statistical Computing). Categorical data are presented as frequencies and percentages, with differences between the DR and non-DR groups assessed using chi-square tests, Mann-Whitney *U* tests, and Kruskal-Wallis tests.

Generalized estimating equations (GEEs) with a binomial distribution and logit link function were used to investigate the association between AL and the presence of tessellated fundus (TF). An exchangeable working correlation structure was specified to account for the correlation between two eyes of the same individual, and robust (sandwich) SEs were used. The analysis was performed separately for the DR group (556 eyes) and the non-DR group (2764 eyes).

For variables with 5% to 15% missing values (mainly AL), multiple imputation by chained equations (10 imputations and 10 iterations) was performed in R. A δ-adjustment sensitivity analysis (delta range –1 to 1; step 0.2) was conducted to assess the robustness of the results under different missing-not-at-random assumptions; the findings remained stable. The results of the GEEs, including the coefficient (B), SE, Wald chi-square, odds ratio (OR), 95% CI, and *P* value, are presented in Table S1 in Multimedia Appendix 1. Sensitivity analysis results are shown in Figure 1.

SPSS was used to calculate sensitivity, specificity, and their 95% CI for each subgroup.

**Figure 1.**
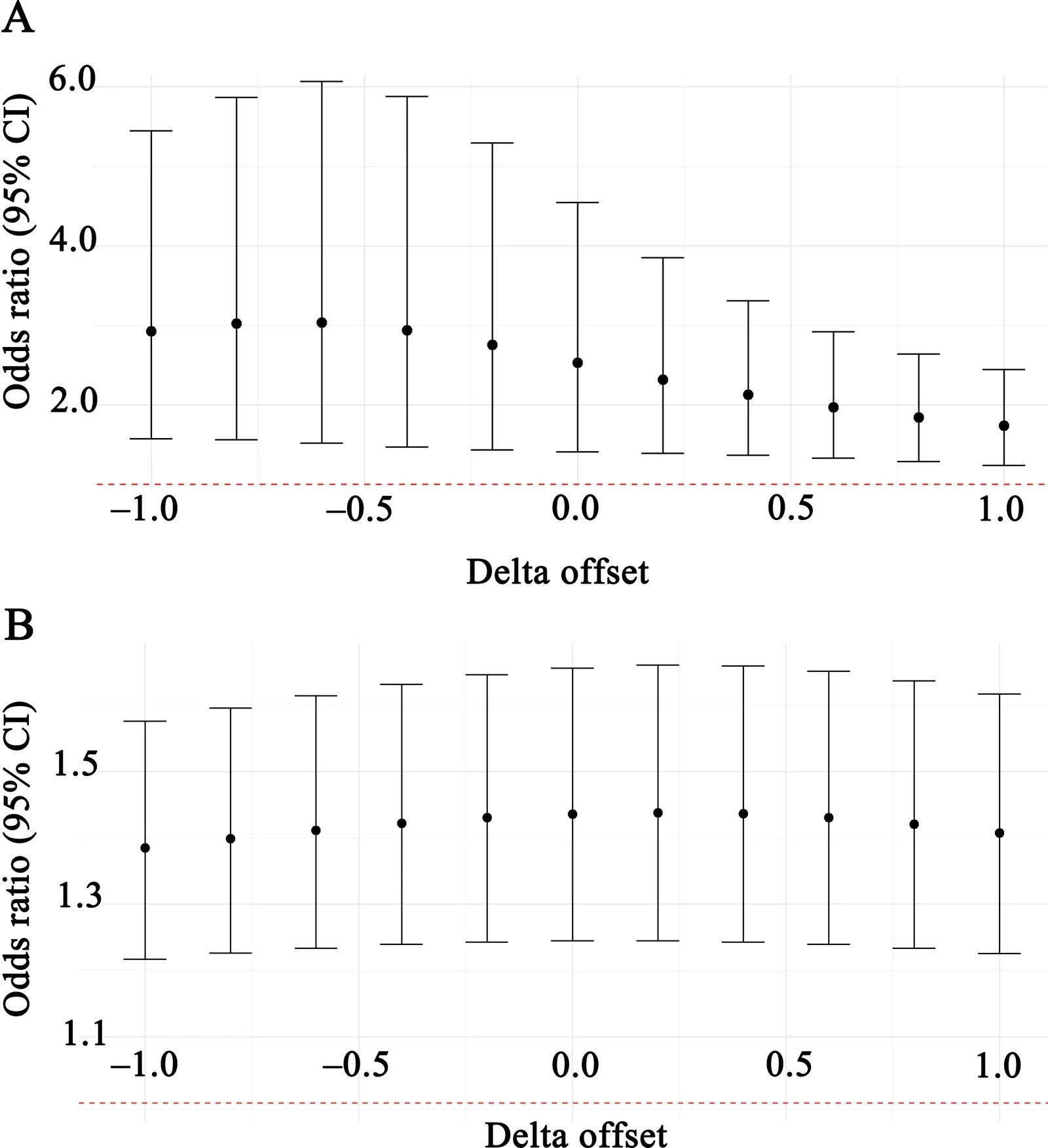
Sensitivity analysis of the association between axial length and severe tessellated fundus conditions under different missing-not-at-random assumptions.

### Ethical Considerations

This study adhered to the principles of the Declaration of Helsinki and was approved by the Ethics Committee of the Affiliated Hospital of Nantong University (2024-K048-01). Data were collected anonymously to protect participants’ confidentiality, and only the research team had access to the dataset. All participants were fully informed before the investigation and signed written informed consent forms; no form of compensation was provided to the participants.

## Results

### AI-Assisted Automated Image Reading Demonstrates Diagnostic Performance Comparable to Human Graders

From October 21 to November 12, 2024, 1847 patients with diabetes (3694 eyes) met the inclusion criteria. Of these, 1748 (94.6%) patients (n=3496, 94.6% eyes) completed the ocular screening according to the protocol (Figure 2). A total of 99 (5.4%) patients were excluded due to various reasons for not completing the 2-field imaging required for analysis by the EVisionAI system. Among the eyes that completed screening with 2-field imaging, 104 (3.0%) eyes were excluded due to poor image quality (eg, resolution too low for discernment or significant eye position deviation affecting DR grading). Ultimately, 3392 (97.0%) eyes were eligible for DR analysis.

The mean age of participants was 67.17 (SD 8.52) years, with a mean diabetes duration of 9.13 (SD 6.97) years. Complete disease characteristics are presented in Table 1. Among all 1748 participants, DR was identified in 335 (19.2%) participants. Of these, 129 (38.5%) had VTDR (7.4% overall prevalence). The EVisionAI demonstrated an overall sensitivity of 90.61% (95% CI 87.89%‐92.79%) and specificity of 98.99% (95% CI 98.52%‐99.31%) for detecting any DR. While its sensitivity was substantially lower in early-stage DR, EVisionAI exhibited diagnostic performance nearly identical to that of human graders in referable cases (such as VTDR). Concurrently, pupillary dilation improved EVisionAI’s sensitivity for DR diagnosis (except in early-stage DR) but had minimal effect on specificity (Table 2).

**Figure 2.**
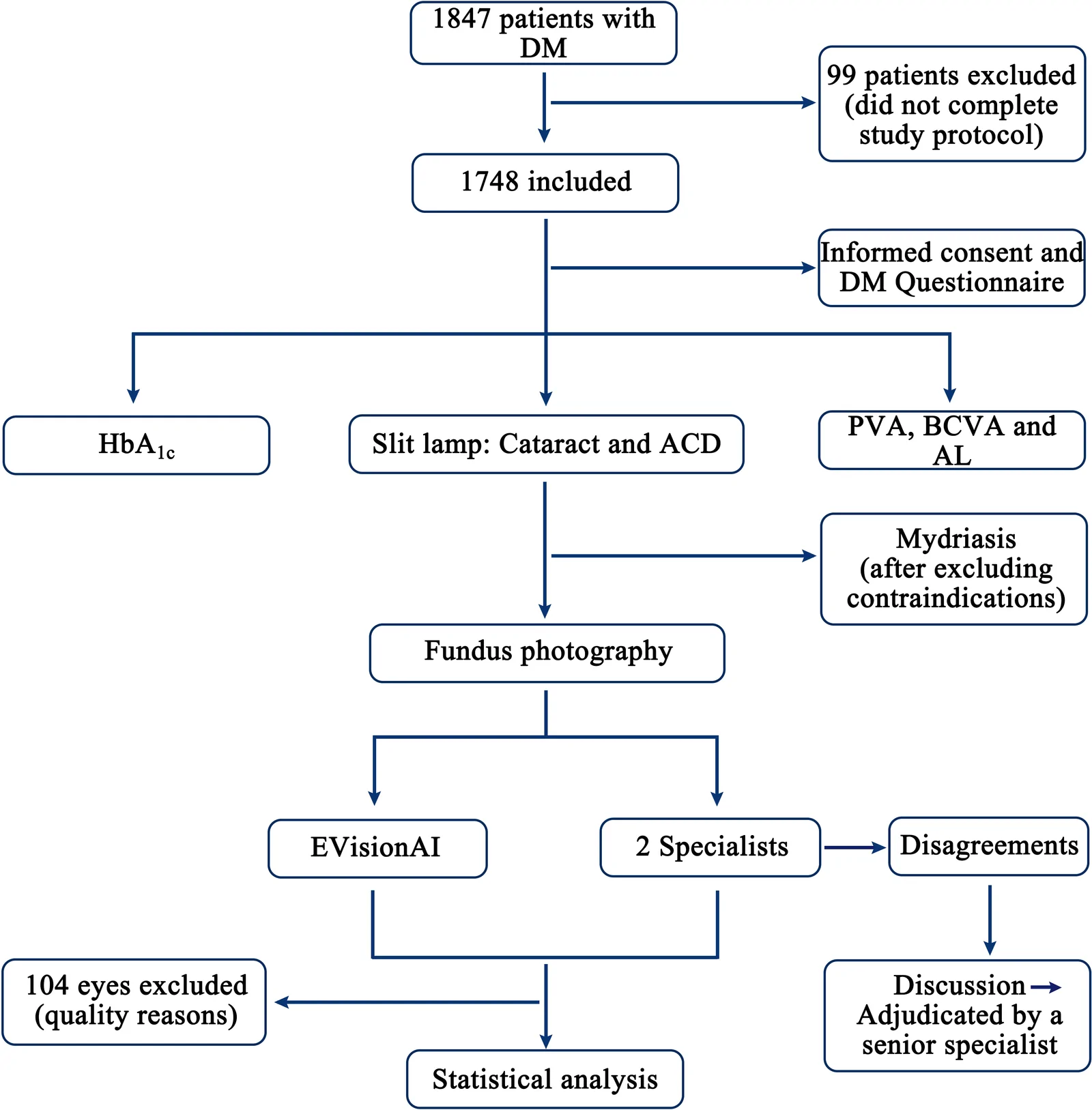
Study procedures in the cross-sectional diagnostic study of the EVisionAI automated diabetic retinopathy detection system. ACD: anterior chamber depth; AL: axial length; BCVA: best-corrected visual acuity; DM: diabetes mellitus; HbA_1c_: hemoglobin A_1c_; PVA: presenting visual acuity.

**Table 1.** Demographic characteristics of participants with and without diabetic retinopathy (DR).

Groups	DR (n=335)	Non-DR (n=1413)	Values	*P* value
Sex, n (%)			0.944^a^	.36
Male	153 (45.7)	604 (42.7)		
Female	182 (54.3)	809 (57.3)		
Age, median (IQR)	66.00 (11.00)	68.00 (12.00)	−3.567^b^	<.001
Duration (y)	13.00 (10.00)	6.00 (8.00)	−11.898^b^	<.001
HbA_1c_ (mmol/mol)	8.70 (2.50)	7.60 (2.20)	−10.131^b^	<.001

a Chi-square test. All chi‑square values have 1 degree of freedom.

b Mann-Whitney *U* test.

**Table 2.** Diagnostic performance of EVisionAI for diabetic retinopathy (DR) under dilated and nondilated conditions.

DR category and mydriasis status	Sensitivity, % (95% CI)	Specificity, % (95% CI)
DR^a^	90.61 (87.89‐92.79)	98.99 (98.52‐99.31)
Dilated	91.37 (88.30‐93.72)	98.79 (98.17‐99.20)
Nondilated	87.41 (80.33‐92.28)	99.23 (98.25‐99.69)
VTDR^b,c^	97.45 (93.82‐99.06)	98.95 (98.51‐99.27)
Dilated	98.13 (94.19‐99.51)	98.81 (98.25‐99.20)
Nondilated	94.45 (79.99‐99.03)	99.32 (98.45‐99.72)
esDR^d,e^	78.46 (73.98‐82.37)	99.19 (98.78‐99.47)
Dilated	78.42 (73.17‐82.91)	99.11 (98.59‐99.45)
Nondilated	78.57 (68.90‐85.96)	99.39 (98.50‐99.78)

a Values in the DR row were calculated using results from all examined participants, regardless of mydriasis status.

b VTDR: vision-threatening diabetic retinopathy.

c Values in the VTDR row were calculated using results from all examined participants, regardless of mydriasis status.

d esDR: early-stage diabetic retinopathy.

e Values in the esDR row were calculated using results from all examined participants, regardless of mydriasis status.

### The Impact of Different Image Clarity on AI Diagnostic Efficacy

To evaluate the impact of refractive media opacity in 2-field imaging on EVisionAI’s diagnostic performance, images were graded based on optic disc and macular clarity according to previous literature [16] and the average values were obtained (Table S2 in Multimedia Appendix 1).

### Impact of Image Clarity Abnormalities on AI Diagnostic Performance at Different DR Stages

The results showed that mild to moderate image clarity impairment, whether in the optic disc or macular region, did not affect EVisionAI’s diagnostic performance. However, severely compromised image clarity resulted in a modest reduction in EVisionAI’s diagnostic sensitivity, while specificity remained largely unaffected (Figure 3 and Table S3 in Multimedia Appendix 1). Notably, for overall DR, reduced clarity in the optic disc region appeared to cause more severe consequences (Figure 3A and Table S3 in Multimedia Appendix 1). In the least clear optic disc cohort (P3 group), sensitivity dropped to 80.95%, indicating potential missed diagnoses within this subset. Conversely, the impact of severely reduced macular clarity on sensitivity was relatively limited (sensitivity in the M3 group only decreased to 87.50%; Figure 3A and Table S3 in Multimedia Appendix 1). Further analysis revealed that the detection rate for early-stage DR was higher in the M3 group than in the P3 group (Table S3 and Table S4 in Multimedia Appendix 1).

**Figure 3.**
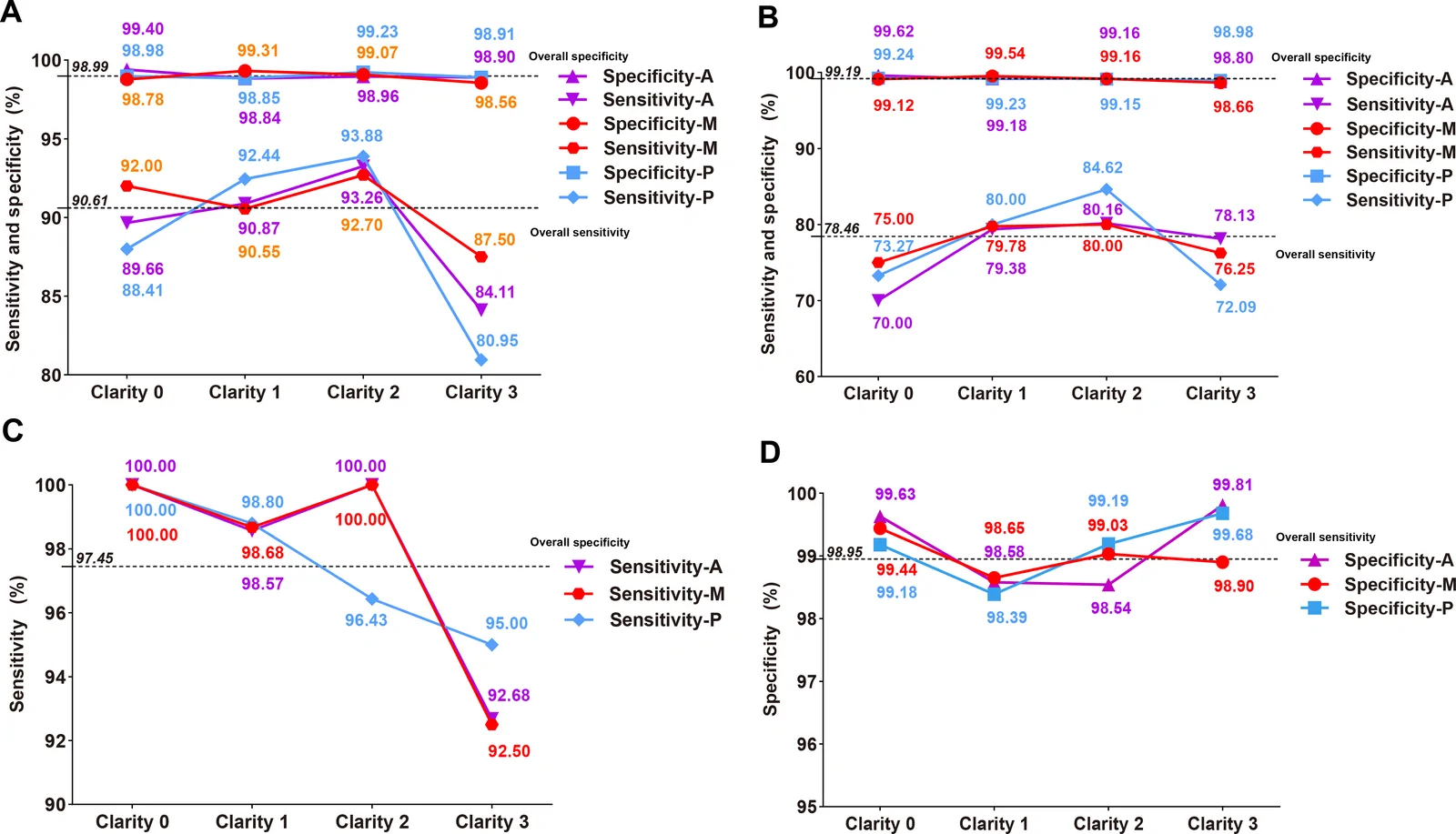
Diagnostic performance of EVisionAI under varying image clarity levels. (A) Specificity and sensitivity of overall diabetic retinopathy (DR) under varying conditions. (B) Specificity and sensitivity of early-stage DR (nonproliferative diabetic retinopathy 1‐2) under varying conditions. (C) Specificity of vision-threatening diabetic retinopathy (VTDR) under varying conditions. (D) Sensitivity of VTDR under varying conditions. A: average clarity of macula and optic disc regions; M: macular region; P: optic disc region.

In the VTDR subgroup, although severe image clarity impairment similarly affected the correct identification of DR, we observed that EVisionAI demonstrated diagnostic capabilities remarkably consistent with those of human graders (Figure 3D and Table S3 in Multimedia Appendix 1). Even within the subgroup with the poorest image clarity (whether in the optic disc or macular region), sensitivity remained above 90%. Further analysis of cases in this subgroup that were not correctly diagnosed (all subgroups where sensitivity failed to reach 100%) revealed that all these cases exhibited severe vitreous degeneration (Figure 4B). This degeneration obscured retinal hemorrhages, preventing their identification, or led to the misidentification of degenerated vitreous as retinal exudates (Figure 4D).

**Figure 4.**
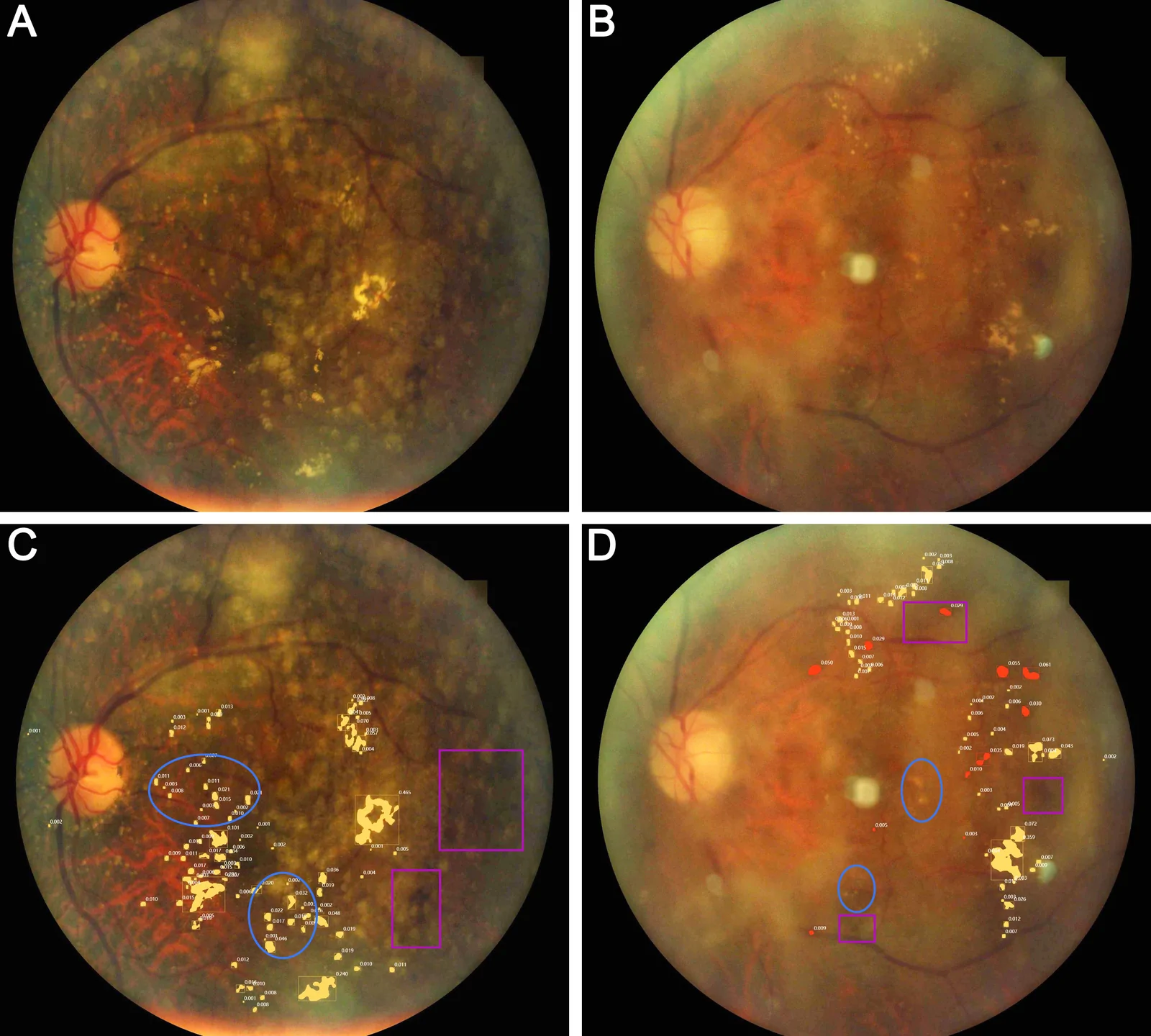
Fundus photography of vitreous degeneration at different clarity levels. (A) Fundus photography of a vitreous degeneration case under M1/P1 clarity and TF3 conditions. (B) Fundus photography of a vitreous degeneration case under M3/P2 clarity and TF1 conditions. (C) Panel A processed by EVisionAI-based automated recognition. (D) Panel B processed by EVisionAI-based automated recognition. Yellow markers: hard exudates; red markers: hemorrhages; blue circle: misidentified exudates (manually added later); magenta box: misidentified hemorrhages (manually added later). The numerical grades (0–3) indicate image quality for M and P, and extent of tigroid fundus for TF, based on the criteria described in the Methods. M: macula‑centered fundus photographs; P: optic disc‑centered fundus photographs; TF: tigroid fundus.

It is noteworthy that for early-stage DR, EVisionAI demonstrated very high specificity while sensitivity was maintained at only around 80% (Figure 3B). Severe defects in image clarity, particularly in the optic disc region, led to a further reduction in sensitivity (Figure 3B and Table S4 in Multimedia Appendix 1). We observed a higher proportion of TF in early-stage DR compared with the VTDR subgroup (VTDR: 52.17% and early DR: 62.24%). To clarify the impact of TF on DR diagnosis, particularly in early DR, we graded the severity of TF in participants according to the reported grading standards [24,25] (Table S2 in Multimedia Appendix 1).

### Impact of Severe TF on AI Diagnostic Performance in Early-Stage DR

Overall, similar to image clarity, severe TF caused a significant decrease in sensitivity, while specificity remained almost unaffected (Figure 5A). In the TF 3 group, sensitivity remained below 85% regardless of whether pupil dilation was performed, indicating a certain probability of missed diagnoses. Further analysis showed that in the TF 3 group, EVisionAI had a relatively low detection rate for early-stage DR (Table 3). Consistent with this, within the early-stage DR subgroup, severe TF led to a sharp decline in sensitivity (Figure 5B), which could not be compensated for even with pupil dilation.

**Figure 5.**
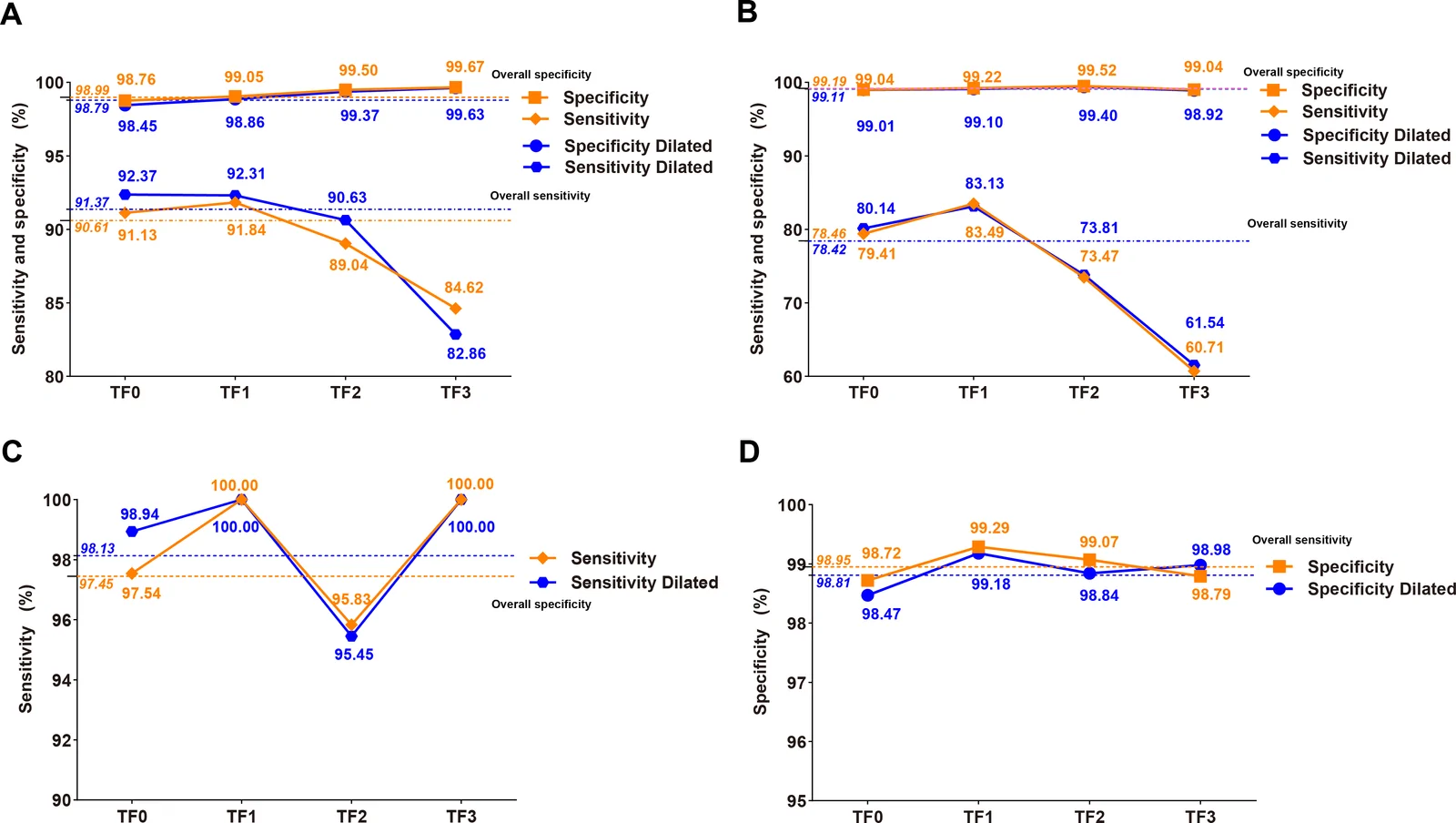
Diagnostic performance of EVisionAI under varying tessellated fundus (TF) changes. (A) Specificity and sensitivity of overall diabetic retinopathy (DR) under varying conditions. (B) Specificity and sensitivity of early-stage DR (nonproliferative diabetic retinopathy [NPDR] 1-2) under varying conditions. (C) Specificity of vision-threatening diabetic retinopathy (VTDR) under varying conditions. (D) Sensitivity of VTDR under varying conditions.

**Table 3.** Detection rate of EVisionAI for different stages of diabetic retinopathy under varying image clarity and degrees of tessellated fundus (TF).

Characteristics	Early-stage DR, n/N (%)	Vision-threatening diabetic retinopathy, n/N (%)
TF 0‐2	289/362 (79.8)	180/184 (97.8)
TF 3	17/28 (60.7)	11/11 (100)
Clarity 0-2^a^	256/326 (78.5)	153/154 (99.4)
Clarity 3^a^	50/65 (76.9)	38/41 (92.7)

a Average clarity of macula and optic disc regions.

### Impact of Severe Vitreous Degeneration on AI Diagnostic Performance in VTDR

For the VTDR subgroup, similar to the image clarity, severe TF changes did not affect EVisionAI’s diagnostic capability (Figure 5D). Further analysis of subgroups where sensitivity did not reach 100% revealed that all patients with misgraded images exhibited severe vitreous degeneration, with some overlapping with patients in the image clarity subgroup (Figure 4). Notably, the vast majority of patients with VTDR whose images were misgraded by EVisionAI simultaneously presented with severe TF changes, image clarity abnormalities, and severe vitreous degeneration. The combination of these factors will undoubtedly significantly compromise both image acquisition quality and EVisionAI’s ability to accurately identify retinal pathology.

## Discussion

### Principal Findings

In this cross-sectional study, EVisionAI was used for automated DR screening among people with diabetes in resource-limited regions in primary health care centers. Although EVisionAI achieved high sensitivity and specificity comparable to those of human graders and successfully identified nearly all VTDR cases, severe ocular factors could still diminish its diagnostic performance, particularly in the detection of early-stage DR. Given its advantages of low cost and high efficiency, as well as the potential optimization of the factors affecting its performance, EVisionAI still represents a cost-effective solution capable of replacing traditional manual grading–dependent DR screening models. Our study identified specific ocular factors that might influence EVisionAI’s diagnostic performance. Optimizing screening protocols for patients exhibiting these factors can significantly enhance recognition efficiency and contribute to further improving the accuracy and referral efficiency for DR in primary health care centers with limited specialists.

### Comparison With Other Studies and Potential Mechanisms

Our research indicated that the accurate identification of early-stage DR was significantly influenced by ocular factors, particularly the extent of TF changes. Subtle lesions, such as microaneurysms and dot hemorrhages, were highly susceptible to being obscured and confused by turbid refractive media and visible choroidal vessels. Consequently, patients at this stage were prone to being missed. From the perspective of diabetes management, screening for mild DR holds substantial clinical value. It enables more patients at this stage to receive earlier medical intervention and personalized diabetes management, thereby reducing or delaying the likelihood of permanent visual impairment [26,27]. Most existing deep learning systems for DR screening primarily focus on the accurate identification of referable DR. Accurately detecting very early-stage DR lesions, such as microaneurysms, remains challenging for most of these systems [11].

Severe TF is typically associated with longer AL and advanced age (Table S1 in Multimedia Appendix 1), with longer AL being a well-established protective factor against the development of DR [28]. Patients who nonetheless progress to VTDR under these conditions generally exhibit poorer glycemic control (Table S5 in Multimedia Appendix 1 and Figure 6), which results in more severe lens opacity and shallower ACD (precluding effective pupil dilation). A cloudy posterior lens capsule and constricted pupil size significantly reduce the clarity of fundus images (Figure 7). Therefore, for older patients with poor glycemic control who concurrently present with these compounding negative factors, meticulous manual re-examination of images can help identify obscured or missed lesions, serving as a valuable compensatory measure. This approach can mitigate the limitations of deep learning systems in these specific scenarios and effectively improve the detection of early-stage DR.

**Figure 6.**
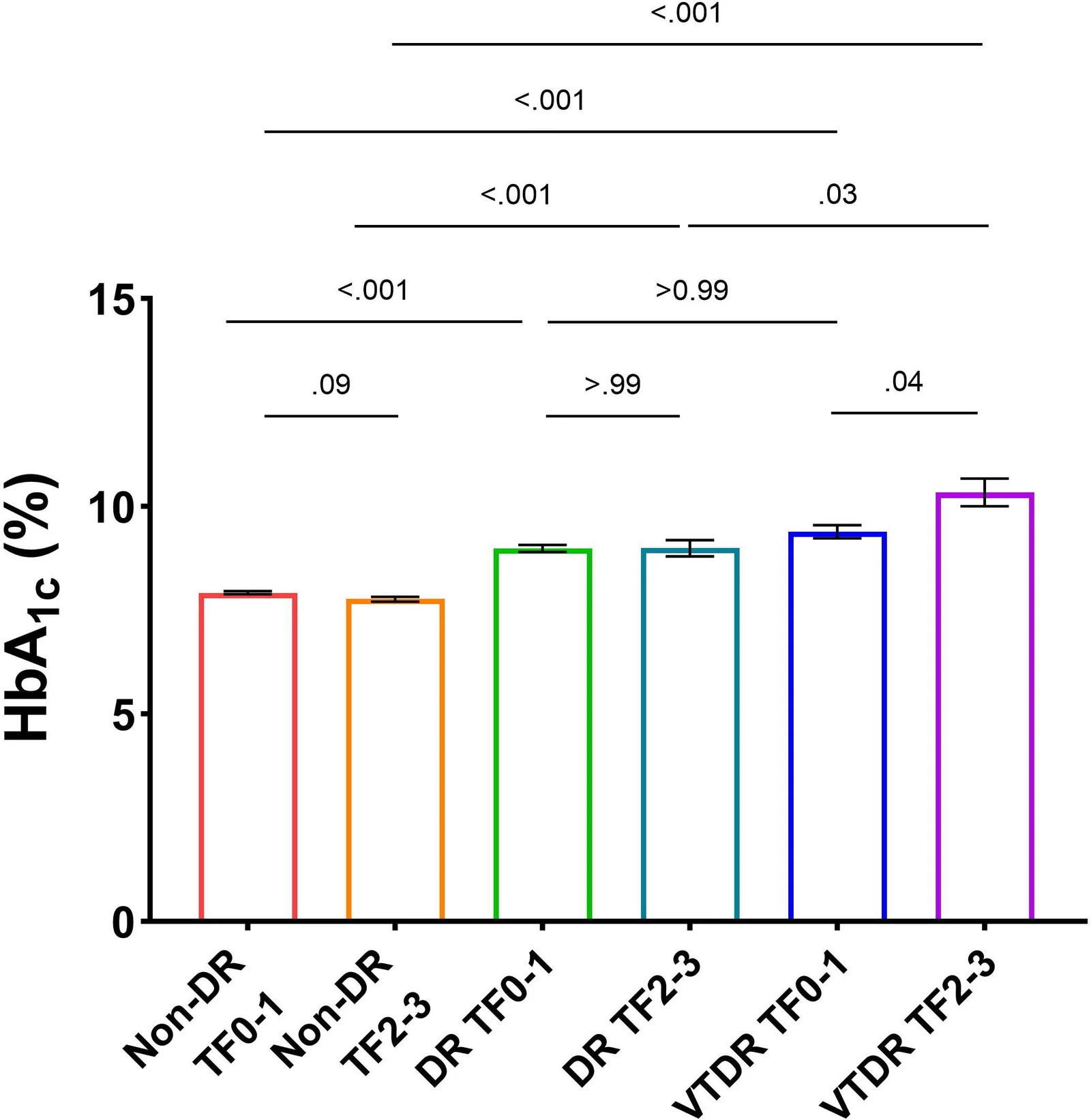
Hemoglobin A_1c_ (HbA_1c_) in diabetic retinopathy (DR) and non-DR participants under different tessellated fundus (TF) conditions. VTDR: vision-threatening diabetic retinopathy.

The decline in image clarity and the presence of TF changes, even when quite severe, almost never affected the correct identification and grading of patients with VTDR who required referral. It is worth noting that while pupil size was related to image quality, it did not significantly impact the correct identification of DR, especially VTDR. In large-scale DR screening conducted at primary health centers without specialized ophthalmologists, pupil dilation may carry significant risks, potentially causing transient increases in intraocular pressure or triggering acute glaucoma attacks. This is particularly critical for participants with severe diabetic optic neuropathy, as a sharp rise in intraocular pressure could cause irreversible vision impairment. In addition, the simplicity of the screening procedure is paramount in primary health care centers, where operators may include technicians and staff without specialized expertise. Our results demonstrated that screening under natural pupil size was already sufficient for the correct identification of DR by deep learning systems. We also acknowledge that our conservative mydriasis criterion (ACD <1/2 CT [corneal thickness]) was adopted to balance participant safety and screening efficiency in rural township settings. This threshold reduced the proportion of dilated participants, which may have confounded the observed effect of severe media opacity on AI performance. Nonetheless, the final image clarity input to the AI reflects real-world nonmydriatic conditions, and our main findings on ocular factors remain valid within this context.

**Figure 7.**
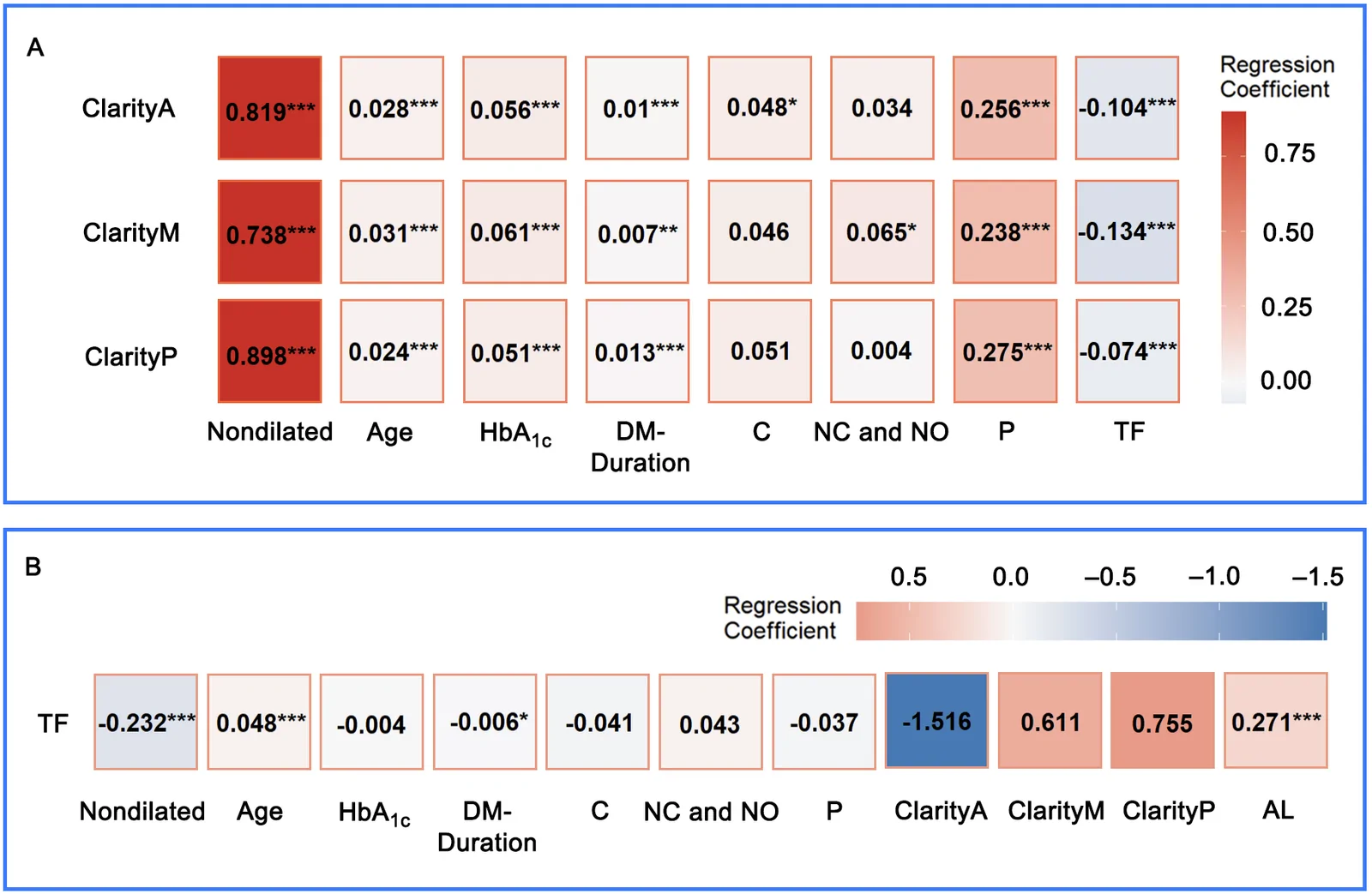
Factors influencing image clarity and tessellated fundus (TF) changes. AL: axial length; C: cortical cataract (LOCS III grade); ClarityA: mean clarity score of the optic disc‑centered (P) and macula‑centered (M) images; ClarityM: clarity of macula‑centered fundus photographs; ClarityP: clarity of optic disc‑centered fundus photographs; DM: diabetes mellitus; NC and NO: nuclear cataract (LOCS III grade); P: posterior subcapsular cataract (LOCS III grade). *Asterisk indicates statistical significance: *P<.05, **P<.01, and ***P<.001.

Severe vitreous degeneration appeared to invariably hinder the accurate identification of retinopathy, even in cases where the retinopathy itself was relatively severe. The markedly turbid vitreous compromised correct image focusing, obscured hemorrhagic lesions, and was mistakenly identified as hard exudates. This interference was particularly pronounced when coexisting with other ocular confounding factors (such as severe image quality deficiency or significant TF). Severe vitreous opacity often indicates either previous vitreous hemorrhage or metabolic abnormalities resulting from prolonged poor glycemic control, both of which indicate a high likelihood of severe DR. Notably, even with excellent image clarity, misdiagnosis primarily caused by severe vitreous degeneration manifested as undergrading rather than overdiagnosis, which undoubtedly led to underestimation of patients with DR who require referral. While cost-effective, the primary goal of community health screening is to efficiently identify patients needing further referral. Therefore, for patients with diabetes presenting with multiple ocular confounding factors—especially severe vitreous degeneration and opacities—existing deep learning systems may not yet fully replace experienced professionals. For this group of patients, we recommend definitive diagnosis by a professional ophthalmologist.

### Meaning of the Study, Strengths, and Limitations

Most previous studies on AI-assisted DR screening focus on diagnostic performance evaluation, with limited analysis of complex ocular confounding factors. Our study provides a detailed analysis of how retinal image clarity (simultaneously affected by refractive media opacity and pupil size) and the degree of TF changes impact AI diagnostic performance. Our findings offer optimization pathways to enhance AI systems’ recognition of DR—particularly early-stage DR with multiple ocular influencing factors—in large-scale screening.

We acknowledge that limitations due to confounding from other social, economic, and cultural factors cannot be entirely excluded. Our sample was recruited from patients with type 2 diabetes registered at the CDC in an economically resource-limited region. Participants were primarily middle-aged and older individuals with lower education levels from rural areas. Although we used stratified sampling as extensively as possible to cover nearly all townships in the local area, compared with the broader general population in China, our sample may exhibit certain differences from regions with lower rural residency and higher education levels. Additionally, our study used a single, albeit well-validated and widely used AI system for DR screening. The lack of cross-validation with other AI models (including commercially available or open-source systems) limits the direct generalizability of our findings to other AI algorithms. Future studies should deliberately include multiple AI systems in the same population to assess whether the identified ocular factors consistently influence diagnostic performance across different AI platforms.

In summary, our study identifies ocular factors affecting deep learning systems’ diagnostic performance in DR screening for patients with type 2 diabetes in resource-limited regions and proposes actionable optimizations. While generalizability to broader populations and other deep learning systems requires further validation, this work enhances DR diagnostic accuracy and referral efficiency by primary care providers in underserved areas, alleviating critical pressures on China’s DR screening programs. This approach expands essential primary health care access while reallocating resources to other medical domains. Future studies will validate findings across diverse populations, health care settings, and more universally applicable AI systems.

## Supplementary material

10.2196/85181 Multimedia Appendix 1 Supplementary figures, tables, AI training details, and references.

## Abbreviations

AAO: American Academy of Ophthalmology
ACD: anterior chamber depth
AL: axial length
BCVA: best-corrected visual acuity
CDC: Center for Disease Control and Prevention
CT: corneal thickness
DR: diabetic retinopathy
GEE: generalized estimating equation
HbA_1c_: hemoglobin A_1c_
NPDR: nonproliferative diabetic retinopathy
PVA: presenting visual acuity
TF: tessellated fundus
VTDR: vision-threatening diabetic retinopathy

## References

[1] Li JP, Liu H, Ting DS (2021). Digital technology, tele-medicine and artificial intelligence in ophthalmology: a global perspective. Prog Retin Eye Res.

[2] Sun H, Saeedi P, Karuranga S (2022). IDF Diabetes Atlas: global, regional and country-level diabetes prevalence estimates for 2021 and projections for 2045. Diabetes Res Clin Pract.

[3] Gurudas S, Vasconcelos JC, Prevost AT (2024). National prevalence of vision impairment and blindness and associated risk factors in adults aged 40 years and older with known or undiagnosed diabetes: results from the SMART-India cross-sectional study. Lancet Glob Health.

[4] Tufail A, Rudisill C, Egan C (2017). Automated diabetic retinopathy image assessment software: diagnostic accuracy and cost-effectiveness compared with human graders. Ophthalmology.

[5] The Lancet Diabetes Endocrinology (2020). Under the lens: diabetic retinopathy. Lancet Diabetes Endocrinol.

[6] Yau JW, Rogers SL, Kawasaki R (2012). Global prevalence and major risk factors of diabetic retinopathy. Diabetes Care.

[7] Wang LZ, Cheung CY, Tapp RJ (2017). Availability and variability in guidelines on diabetic retinopathy screening in Asian countries. Br J Ophthalmol.

[8] Ipp E, Liljenquist D, Bode B (2021). Pivotal evaluation of an artificial intelligence system for autonomous detection of referrable and vision-threatening diabetic retinopathy. JAMA Netw Open.

[9] Shi Q, Zhao Y, Fonseca V, Krousel-Wood M, Shi L (2014). Racial disparity of eye examinations among the U.S. working-age population with diabetes: 2002-2009. Diabetes Care.

[10] Ting DS, Cheung GC, Wong TY (2016). Diabetic retinopathy: global prevalence, major risk factors, screening practices and public health challenges: a review. Clin Exp Ophthalmol.

[11] Dai L, Wu L, Li H (2021). A deep learning system for detecting diabetic retinopathy across the disease spectrum. Nat Commun.

[12] (2021). IDF Diabetes Atlas.

[13] Hu C, Jia W (2018). Diabetes in China: epidemiology and genetic risk factors and their clinical utility in personalized medication. Diabetes.

[14] Abràmoff MD, Lavin PT, Birch M, Shah N, Folk JC (2018). Pivotal trial of an autonomous AI-based diagnostic system for detection of diabetic retinopathy in primary care offices. NPJ Digit Med.

[15] Gargeya R, Leng T (2017). Automated identification of diabetic retinopathy using deep learning. Ophthalmology.

[16] Gulshan V, Peng L, Coram M (2016). Development and validation of a deep learning algorithm for detection of diabetic retinopathy in retinal fundus photographs. JAMA.

[17] Ting DS, Cheung CY, Lim G (2017). Development and validation of a deep learning system for diabetic retinopathy and related eye diseases using retinal images from multiethnic populations with diabetes. JAMA.

[18] Bhaskaranand M, Ramachandra C, Bhat S (2019). The value of automated diabetic retinopathy screening with the EyeArt system: a study of more than 100,000 consecutive encounters from people with diabetes. Diabetes Technol Ther.

[19] Wang Y, Yu M, Hu B (2021). Deep learning‐based detection and stage grading for optimising diagnosis of diabetic retinopathy. Diabetes Metab Res Rev.

[20] Rajesh AE, Davidson OQ, Lee CS, Lee AY (2023). Artificial intelligence and diabetic retinopathy: AI framework, prospective studies, head-to-head validation, and cost-effectiveness. Diabetes Care.

[21] Wang M, Zhou X, Liu DN, Chen J, Zheng Z, Ling S (2022). Development and validation of a predictive risk model based on retinal geometry for an early assessment of diabetic retinopathy. Front Endocrinol (Lausanne).

[22] He HL, Liu YX, Liu H (2024). Deep learning-enabled vasculometry depicts phased lesion patterns in high myopia progression. Asia Pac J Ophthalmol (Phila).

[23] (2016). Diabetic retinopathy - Asia. American Academy of Ophthalmology.

[24] Guo Y, Liu L, Zheng D (2019). Prevalence and associations of fundus tessellation among junior students from Greater Beijing. Invest Ophthalmol Vis Sci.

[25] Yan YN, Wang YX, Xu L, Xu J, Wei WB, Jonas JB (2015). Fundus tessellation: prevalence and associated factors: the Beijing Eye Study 2011. Ophthalmology.

[26] Flaxel CJ, Adelman RA, Bailey ST (2020). Diabetic retinopathy preferred practice pattern®. Ophthalmology.

[27] American Diabetes Association (2020). 11. Microvascular complications and foot care: *Standards of Medical Care in Diabetes-2020*. Diabetes Care.

[28] Li Y, Hu P, Li L, Wu X, Wang X, Peng Y (2024). The relationship between refractive error and the risk of diabetic retinopathy: a systematic review and meta-analysis. Front Med (Lausanne).

