# Supplementary material for: Ocular Factors Affecting AI Diagnosis in Diabetic Retinopathy Screening for Resource-Limited Regions: Cross-Sectional Study
Source: J Med Internet Res. 2026 Sep 30;28:e85181. doi: 10.2196/85181 (PMC13626077; doi:10.2196/85181)
Supplement: Supplementary file 1 [file jmir-v28-e85181-s001.doc]

**Table S1** Characteristics of AL and Age in DR and non-DR Participants under Different TF Conditions.

| Group | TF 0-2 | TF 3 | *B*/*U^c^* | *SE* | *Wald χ²* | *OR* | *95% CI* | *P* |
| --- | --- | --- | --- | --- | --- | --- | --- | --- |
| **DR AL (mm)^a^** | 22.98±0.76 | 23.57±0.49 | 0.93 | 0.29 | 19.33 | 2.53 | 1.41-4.55 | .0018 |
| **Non-DR AL (mm)^a^** | 23.05 (1.07) | 23.60 (0.89) | 0.36 | 0.07 | 22.89 | 1.44 | 1.25-1.66 | <.001 |
| **DR Age (y)^b^** | 66.00 (12.00) | 71.00 (6.00) | -5.15 | - | - | - | - | <.001 |
| **Non-DR Age (y)^b^** | 67.00 (12.00) | 73.00 (9.00) | -11.93 | - | - | - | - | <.001 |

^a^Generalized Estimating Equations (GEE); ^b^Mann-Whitney *U* test.

*^c^B*, regression coefficient from generalized estimating equations (GEE); *U*, Mann-Whitney U statistic.

**Table S2 Number of Eyes in Each Group.**

|  | **Clarity A** | **Clarity M** | **Clarity P** | **TF** | **TF & Dilated** |
| --- | --- | --- | --- | --- | --- |
| **0** | 560 | 733 | 1018 | 1490 | 953 |
| **1** | 1264 | 1265 | 1197 | 886 | 641 |
| **2** | 961 | 678 | 796 | 673 | 540 |
| **3** | 607 | 716 | 381 | 343 | 304 |

Clarity A/M/P: A: Average clarity of macula and optic disc regions; M: Macular region, P: Optic disc region;

TF: Tessellated fundus changes; TF & Dilated: Tessellated fundus changes under dilated conditions.

**Table S3 Diagnostic Performance of EVisionAI under varying conditions.**

| **Grade** |  | **Clarity A0** | **Clarity A1** | **Clarity A2** | **Clarity A3** | **Clarity M 0** | **Clarity M 1** | **Clarity M 2** | **Clarity M 3** | **Clarity P 0** | **Clarity P 1** | **Clarity P 2** | **Clarity P 3** | **TF 0** | **TF 1** | **TF 2** | **TF 3** | **TF 0 Dilated** | **TF 1 Dilated** | **TF 2 Dilated** | **TF 3 Dilated** |
| --- | --- | --- | --- | --- | --- | --- | --- | --- | --- | --- | --- | --- | --- | --- | --- | --- | --- | --- | --- | --- | --- |
| **DR** | **Sensitivity**  **(%)** | 89.66 | 90.87 | 93.26 | 84.11 | 92.00 | 90.55 | 92.70 | 87.50 | 88.41 | 92.44 | 93.88 | 80.95 | 91.13 | 91.84 | 89.04 | 84.62 | 92.37 | 92.31 | 90.63 | 82.86 |
|  | **95 CI**  **(%)** | 78.16-  95.72 | 86.19-  94.13 | 88.51-  96.22 | 75.50-  90.21 | 82.79-  96.71 | 86.10-  93.73 | 86.64-  96.25 | 79.92-  92.60 | 81.58-  93.02 | 88.12-  95.33 | 88.35-  96.98 | 68.71-  89.36 | 87.38-  93.88 | 85.86-  95.52 | 79.02-  94.81 | 68.79-  93.59 | 88.02-  95.29 | 85.50-  96.20 | 80.05-  96.13 | 65.70-  92.83 |
|  | **Specificity**  **(%)** | 99.40 | 98.84 | 98.96 | 98.90 | 98.78 | 99.31 | 99.07 | 98.56 | 98.98 | 98.85 | 99.23 | 98.91 | 98.76 | 99.05 | 99.50 | 99.67 | 98.45 | 98.86 | 99.37 | 99.63 |
|  | **95 CI**  **(%)** | 98.11-  99.85 | 97.92-  99.37 | 97.87-  99.51 | 97.31-  99.60 | 97.52-  99.43 | 98.51-  99.70 | 97.71-  99.66 | 97.06-  99.33 | 97.99-  99.50 | 97.89-  99.40 | 98.10-  99.72 | 96.57-  99.72 | 97.87-  99.29 | 97.96-  99.58 | 98.41-  99.87 | 97.88-  99.98 | 97.15-  99.18 | 97.40-  99.53 | 98.01-  99.84 | 97.62-  99.98 |
| **esDR (NPDR**  **1-2)** | **Sensitivity**  **(%)** | 70.00 | 79.38 | 80.16 | 78.13 | 75.00 | 79.78 | 80.00 | 76.25 | 73.27 | 80.00 | 84.62 | 72.09 | 79.41 | 83.49 | 73.47 | 60.71 | 80.14 | 83.13 | 73.81 | 61.54 |
|  | **95 CI**  **(%)** | 53.29-  82.91 | 72.11-  85.19 | 71.91-  86.52 | 65.71-  87.11 | 60.77-  85.52 | 72.97-  85.26 | 69.26-  87.80 | 65.19-  84.75 | 63.38-  81.36 | 72.66-  85.82 | 75.19-  91.03 | 56.10-  84.17 | 73.08-  84.61 | 74.89-  89.66 | 58.66-  84.60 | 40.73-  77.87 | 72.41-  86.19 | 72.98-  90.15 | 57.68-  85.61 | 40.73-  79.09 |
|  | **Specificity**  **(%)** | 99.62 | 99.18 | 99.16 | 98.80 | 99.12 | 99.54 | 99.16 | 98.66 | 99.24 | 99.23 | 99.15 | 98.98 | 99.04 | 99.22 | 99.52 | 99.04 | 99.01 | 99.10 | 99.40 | 98.92 |
|  | **95 CI**  **(%)** | 98.46-  99.93 | 98.40-  99.60 | 98.20-  99.63 | 97.27-  99.51 | 90.74-  94.61 | 98.86-  99.83 | 97.93-  99.69 | 97.26-  99.37 | 98.36-  99.67 | 98.43-  99.64 | 98.05-  99.65 | 96.80-  99.74 | 98.28-  99.48 | 98.23-  99.68 | 98.47-  99.88 | 97.00-  99.75 | 97.97-  99.54 | 97.80-  99.67 | 98.09-  99.84 | 96.62-  99.72 |
| **vtDR (NPDR 3 & PDR)** | **Sensitivity**  **(%)** | 100.00 | 98.57 | 100.00 | 92.68 | 100.00 | 98.68 | 100.00 | 92.50 | 100.00 | 98.80 | 96.43 | 95.00 | 97.54 | 100.00 | 95.83 | 100.00 | 98.94 | 100.00 | 95.45 | 100.00 |
|  | **95 CI**  **(%)** | 77.08-  100.00 | 91.23-  99.93 | 93.24-  100.00 | 78.99-  98.09 | 81.50-  100.00 | 91.89-  99.93 | 92.13-  100.00 | 78.52-  98.04 | 87.99-  100.00 | 92.54-  99.94 | 86.62-  99.38 | 73.06-  99.74 | 92.44-  99.36 | 88.57-  100.00 | 76.88-  99.78 | 67.86-  100.00 | 93.38-  99.94 | 87.36-  100.00 | 75.12-  99.76 | 62.88-  100.00 |
|  | **Specificity**  **(%)** | 99.63 | 98.58 | 98.54 | 99.81 | 99.44 | 98.65 | 99.03 | 98.90 | 99.18 | 98.39 | 99.19 | 99.68 | 98.72 | 99.29 | 99.07 | 98.79 | 98.47 | 99.18 | 98.84 | 98.98 |
|  | **95 CI**  **(%)** | 98.52-  99.94 | 97.68-  99.14 | 97.45-  99.19 | 98.77-  99.99 | 98.46-  99.28 | 97.77-  99.20 | 97.78-  99.60 | 97.63-  99.52 | 98.33-  99.62 | 97.41-  99.01 | 98.15-  99.67 | 97.98-  99.98 | 97.92-  99.23 | 98.38-  99.71 | 97.88-  99.62 | 96.72-  99.61 | 97.33-  99.15 | 97.97-  99.70 | 97.36-  99.53 | 96.81-  99.74 |

Clarity A/M/P: A: Average clarity of macula and optic disc regions; M: Macular region, P: Optic disc region; TF: Tessellated fundus changes; TF & Dilated: Tessellated fundus changes under dilated conditions.

**Table** **S4 Detection Rate of EVisionAI for DR under Grade 3 Image Clarity Condition.**

|  | **NPDR 1** | **NPDR 2** | **NPDR 3 + PDR** |
| --- | --- | --- | --- |
| **Clarity M3** | 62.50% (5/8) | 61.64% (45/73) | 92.50% (37/40) |
| **Clarity P3** | 20.00% (1/5) | 57.89% (22/38) | 95.00% (19/20) |

Clarity M/P: M: Macular region, P: Optic disc region.

**Table S5 HbA1c in DR and non-DR Participants under Different TF Conditions.**

|  | **Non-DR** | **DR** | **vtDR** |
| --- | --- | --- | --- |
| **TF 0-1** | 63.06 ±1.0 mmol/mol [7.92± 1.66%] | 74.65 ±1.0 mmol/mol [8.98± 1.84%] | 79.02 ±1.0 mmol/mol [9.38± 2.00%] |
| **TF 2-3** | 59.89 ±1.0 mmol/mol [7.76± 1.68%] | 74.65 ±1.0 mmol/mol [8.98± 2.07%] | 86.23 ±1.0 mmol/mol [10.04± 2.14%] |

**Supplementary Note. 1. Additional details of EVisionAI.**

1. Architecture of the deep learning system

EVisionAI is an integrated intelligent analysis system that combines deep learning networks with conventional computer vision algorithms, inspired by the bionic mechanism of human vision [1,2]. Its core components include:

- Optic disc detection: A Single Shot Detection (SSD) model with ResNet-50 backbone, followed by polar-coordinate edge detection and minimum circumscribed circle fitting [2,3].
- Lesion segmentation (hemorrhages, exudates, microaneurysms): Initially based on mathematical morphology and support vector machines in the earlier SmartEye version [1]; the current EVisionAI employs a ResNet101-UNet architecture for precise segmentation of lesions, peripapillary atrophy (PPA), and retinal vessels [3,4].
- Vessel extraction and classification: ResNet101-UNet for vessel segmentation, with arteriovenous differentiation based on color, brightness, and topological relationships [3,4].
- Image preprocessing and quality assessment: Automated ROI extraction, denoising, normalization, and exposure/clarity evaluation using Fourier transform and local feature analysis [2,5]. This quality module identifies poor-quality images (e.g., due to cataracts, small pupils, or defocus) and excludes them from further analysis [1,5].

2. Training dataset

The system was trained and validated on large-scale fundus image datasets from multiple sources:

- DR grading and lesion detection: Tens of thousands of images from the Shanghai Diabetic Eye Study (SDES) and other community-based screening programs, with annotations by certified ophthalmologists [1].
- Optic disc and PPA segmentation: 4,100 images with manually labeled optic disc bounding boxes [2]; additional 500 validation samples yielded accuracy >0.99 [2,3].
- Vessel segmentation: Training/validation/testing split of 8:1:1, achieving an accuracy of 0.970, sensitivity 0.859, and IoU 0.739 [3].
- The datasets cover various cameras (Canon, Topcon, Zeiss, Newvision) and patient ages (children to elderly), ensuring generalizability [2,6].

3. Input parameters

The primary input is a color fundus photograph (45° field-of-view, macula- or disc-centered) captured by non-mydriatic fundus cameras. The system automatically:

- Detects the region of interest (ROI) and removes black background.
- Normalizes color, brightness, and exposure to a standard reference range.
- Extracts relevant features (optic disc, vessels, lesions) without requiring manual parameter tuning.

Outputs include DR severity grading (based on international clinical DR scale) and quantitative lesion metrics (e.g., total hemorrhage area, exudation area) at pixel level [1,2].

4. Calibration method

The pixel pitch (actual size per pixel in fundus) was calibrated using a generic ROI–optic disc ratio method described in Long et al. [2]. Briefly:

- The system automatically measures the ROI diameter (in pixels) and optic disc diameter (in pixels).
- Based on prior knowledge of the average human optic disc diameter (1.921 ± 0.026 mm from meta-analysis of 40,600 images), the average pixel pitch is calculated as ROI diameter in fundus/ROI diameter in pixels ROI diameter in fundus/ROI diameter in pixels.
- For 45° FOV cameras, the average ROI–disc ratio is 6.404 ± 0.619, enabling direct pixel pitch estimation without camera-specific parameters [2].
- This calibration ensures that measured lesion areas (e.g., hemorrhage in mm²) are consistent across different camera models and have been validated against ISO 10940:2009 with <5% bias [2].

**References**

[1] Xu Y, Wang Y, Liu B, et al. The diagnostic accuracy of an intelligent and automated fundus disease image assessment system with lesion quantitative function (SmartEye) in diabetic patients. BMC Ophthalmol. 2019;19:184.

[2] Long T, Xu Y, Zou H, et al. A generic pixel pitch calibration method for fundus camera via automated ROI extraction. Sensors. 2022;22(21):8565.

[3] He HL, Liu YX, Liu H, et al. Deep learning-enabled vasculometry depicts phased lesion patterns in high myopia progression. Asia-Pac J Ophthalmol. 2024; in press. DOI:10.1016/j.apjo.2024.100086.

[4] Liu F, Yu XH, Wang YC, et al. Quantitative analysis of optic disc changes in school-age children with ametropia based on artificial intelligence. Int J Ophthalmol. 2023;16(11):1727-1733.

[5] Xu Y, Ling SG, Dong Z, et al. Development and application of a fundus image quality assessment system based on computer vision technology. Chin J Ophthalmol. 2020;56(12):920-927.

[6] Peng J, Yao H, Li LJ, et al. Epidemiological survey of high myopia in people aged 18 years or older in Chongqing. Chin J Optom Ophthalmol Vis Sci. 2023;25(10):739-747.
